# Rupture of the Womb—Recovery

**Published:** 1868-10

**Authors:** S. A. McWilliams

**Affiliations:** Chicago


ARTICLE XL.

### RUPTURE OF THE WOMB—RECOVERY.

REPORTED BY S. A. McWILLIAMS, A.M., M.D., Chicago.

Dr. David B. Taylor, Milbourne, Lake Co., Ill., was called May 27, 1868, about 9 A.M., to attend Mrs. Hinkle, a farmer's wife, living one mile distant, in her eighth confinement, at full term. All her previous labors, except the last, were severe.

The lady is of German birth, 40 years old; has given birth twice to twins, and has now five children living.

Upon examination, the Doctor found the os uteri about an inch dilated, and the head presenting. The liquor amnii had escaped about three hours previously. The pains continued light, until 2 P.M., when the os was sufficiently dilated to permit the use of instruments. A severe pain occurred about this time, which caused some progress; in five minutes, a similar one, and soon another, which promised to be still stronger, but suddenly began to die away, while the head gradually receded, and flowing commenced.

The Doctor, satisfied that a rupture had taken place, turned and delivered the head with instruments, unable to do so otherwise. The child—a male—was dead when born, and weighed 8½ lbs. The placenta was found detached, and delivered at once.

A further examination revealed a rupture to the right of the median line, through which the Doctor readily passed his hand, and removed a couple of handfuls of blood, when the womb began to contract rapidly. The woman, being now threatened with syncope, was given three 2-dr. doses of brandy every eight minutes. A Dover's powder being now given, she was allowed to rest 2½ hours. Reaction commenced—pulse 100 per minute. A powder of 3 grs. calomel and ½ gr. opium was ordered to be given every two hours; also, 2 dr. nitrate of potash, to be dissolved in half a tumbler of water, of which a dr. was to be given every hour. During the night, tympanitis set in, became very severe, and lasted about three weeks, subsiding gradually with the diarrhœa. A mush poultice was kept on bowels for the first ten days, when an eruption of vesicles appeared on abdomen.

*May 28, 8 A.M.*—Pulse 125. Continued solution of potash, and ordered 5 gr. calomel and 1 gr. opium every two hours.

*8 P.M.*—Omitted solution of potash, and gave 10 gtts. *til. ex.* gelseminum every four hours for forty-eight.

*29th, 8 A.M.*—Pulse 140. Gave 10 grs. calomel, 1½ gr. opium every three hours for six days, when bowels were moved with an injection of soap-suds.

*June 5th.*—Pulse 150. Bowels moved about eight times daily for the ensuing week, then gradually improved, and she vomited occasionally during the next three weeks.

Previous remedies omitted. 3 gtts. nitro-muriatic acid given every four hours for thirty-six, and ⅓ gr. nitrate of silver in solution every four hours.

*6th.*—Lochial discharge now occurred, for the first time.

After two weeks, a pill of 2 gr. quinia and 1 gr. precipitated carbonate of iron were given every four hours for four or five days, when tympanitis had nearly subsided.

After which, 15 gtts. tr. fierri chlor. and 2 gr. chlorate potass, in syrup, were given four times daily.

The diet consisted mainly of crust, coffee, barley and rice water, for the first two weeks; after which she was able to take bland articles of nourishment.

*July 28.*—The woman is around, attending to her ordinary household duties.

